# Reply to Child, R. Comment on “Cesak et al. Carnosine and Beta-Alanine Supplementation in Human Medicine: Narrative Review and Critical Assessment. *Nutrients* 2023, *15*, 1770”

**DOI:** 10.3390/nu16162575

**Published:** 2024-08-06

**Authors:** Ondrej Cesak, Jitka Vostalova, Ales Vidlar, Petra Bastlova, Vladimir Student

**Affiliations:** 1 Department of Urology, University Hospital Olomouc, 775 20 Olomouc, Czech Republic; ondrej.cesak@fnol.cz (O.C.);; 2 Faculty of Medicine and Dentistry, Palacky University, 775 15 Olomouc, Czech Republic; 3 Department of Medical Chemistry and Biochemistry, Faculty of Medicine and Dentistry, Palacky University, 775 15 Olomouc, Czech Republic; 4 Department of Rehabilitaion, University Hospital Olomouc, 775 20 Olomouc, Czech Republic

Thank you very much for your comment [1]. We really appreciate your interest in our work [2], as carnosine and its precursors beta-alanine and histidine are crucial molecules in human medicine, as presented in the narrative review. 

Increasing the dietary intake of carnosine enhances its concentrations in, mostly, skeletal muscle, the brain, and the heart [3]. For that reason, it is essential to investigate its food sources, and we thank you for raising this issue. 

Asparagus demonstrates high levels of basic nutrients, including vitamins, amino acids, and mineral salts, it is rich in fiber, and it contains large amounts of folic acid and vitamin C [4]. Wu et al. [5] demonstrated that green peas are rich in macronutrients, including proteins, starches, dietary fiber, and non-starch polysaccharides. White mushrooms contain beta-glucans, ergosterol, ergothioneine, vitamin D, and an antioxidant compound usually reported as flavonoids [6]. Many sources [3], as well as your comment, do mention histidine dipeptides never having been found in plants. I find it beneficial to bring attention to a breakthrough paper by Kukreti et al. (2023), published a few months after our narrative review [7]. The authors showed, using LC/MS analysis, that the hydro-alcoholic extract of the plant *Skimmia anquetilia* contains multiple important active constituents, interestingly also including L-carnosine. [7] 

Although asparagus, green peas, and white mushrooms are surely beneficial and important components of a healthy human diet, upon further investigation of the literature, the three above-mentioned food sources showed no carnosine content. 

Crucially, important dietary sources of carnosine include foods such as chicken meat [8], fish, and shrimp [9], as you stated in your comment and as stated in the presented paper. 

All of the above food sources certainly contribute to a healthy diet, and their consumption is, for obvious reasons, recommended.
